# Prosthetic Dentistry

**Published:** 1901-04-15

**Authors:** G. H. Wilson

**Affiliations:** Cleveland, Ohio


### PROSTHETIC DENTISTRY.

BY G. H. WILSON, D.D.S., CLEVELAND, OHIO.

Read at Ohio State Dental Society, December, 1900.

While prosthetic dentistry has been very much discussed and abused in the years gone by, I believe the time is at hand when it will be more rationally considered, and accorded the noble position to which it is entitled.

To-day the profession recognizes four distinct divisions in the practice of stomatology—operative, prosthetic, surgical, and orthopedic dentistry. Each department overlaps one or more of the others, so that the lines of demarkation are arbitrary and not natural. The terms used I believe are the best that have been suggested, but we must recognize that general terms have been applied to specific uses. Operative can, if used in its broadest sense, be made to cover every remedial act in stomatology wherein an instrument or appliance is used. This would only leave diagnosis, prognosis and prescription writing not included in the literal use of the term, while they are an essential part of the meaning implied by our specific use of the word.

Prosthesis means a restoration of, and literally would include all fillings, as well as crowns, bridges, dentures, velums and obturators.

We favor the term prosthesis rather than the new term proposed by Dr. Ottolengui, prosthodontia, because the latter term means restoration of the teeth, while the former is applicable to the restoration of the teeth or parts thereof, also of their associate parts. Prosthodontia is too limited a term, and I believe will tend to restrict our department of practice. I shall endeavor to show by this paper that art in prosthesis requires as much attention given to restoring the soft tissues as to the teeth, and thus incidentally prove that the new term is not as good as the old.

A good rule by which to decide what may be considered operative dentistry and what prosthetic, is in its relation to the operating chair and laboratory bench. If the essential or major portion of an operation is performed at the chair, it is operative dentistry; if the essential or major portion is at the bench, then it should be considered prosthetic. The two remaining branches of stomatology are offshoots of the two fundamental departments under consideration, and need not be further mentioned. There has been some contention as to which department crown and bridge work belong, but now it is generally associated with the prosthetic.

As prominence has recently been given to inlay work, it has caused some discussion as to the department to which it belongs. The executive council of the National Dental Association, at its late session, recommended that it be classed as operative. I demur against the decision of the committee, and believe the profession will in the near future support the protest. Inlay work is in the nature of enameled platinum crowns, and the larger portion of the work is done with laboratory appliances, and, judged by our rule, must be classed as bench work; hence prosthetic.

I know I have few sympathizers when I assert that prosthesis is the greatest and should be the most exalted portion of our profession. By that I do not mean mechanical dentistry, as so commonly practiced; I mean prosthesis, a restoration of the lost tissues, functions and expression, second only to the work of the

Creator, and that because of the substances of which the restorations are made and their inherent limitations. Prosthesis is the greater, because it requires more intelligence, patience, manipulative ability and skill than the operative and is fraught with such dire consequences when improperly understood and practiced. That operative dentistry is the most essential I would as strongly affirm, because it is conservative, and prosthesis must always be handicapped by the inherent defects in the materials and methods used for restorations. The operator combats the destroyer at an earlier stage than the prosthetist, and the larger number of our profession are capable of attaining reasonable success in the operative department. The prosthetic is greater because it must begin where the operative has exhausted its resources. All honor to the noble men who are doing so much for their fellow men through their operative work; but we do believe they have not conceived the importance of, or the possibilities of prosthesis. We ask the aid of our operators in developing this important branch of our profession, by sending patients and not casts to the prosthetist. I can not take time to discuss the causes of the degradation that has befallen prosthetic dentistry in times past.

We deprecate the idea that has been entertained by so many, that there should be a divorcement between the two great divisions of dentistry, and that there should be a special course of training for each. I believe the best interest of the patient will be conserved by the devotees to the dental art and science receiving a broad and liberal education upon the whole subject of stomatology, and, after having pursued general practice for a time, specializing as their tastes and ability may develop. It is only by a course of this kind that the conservative possibilities of the operator can be appreciated by the prosthetist.

It can not be truthfully said that prosthesis is more mechanical than operative dentistry, only that there are greater varieties of mechanical procedure, while it can be said that the talents of the fine arts are almost entirely confined to the prosthetist. He must have some of the sense of color of the painter, much of the modeling and carving of the sculptor, and the sense of proportion and harmony that belongs to all true artists. The prosthetist should be a student of mankind, both of the mind and material body, that he may vitalize and individualize his work.

The desire is to direct attention to the artistic side of prosthesis, but as we can not follow this thought in all divisions of prosthesis without making too lengthy a paper, we will confine our thoughts to full dentures.

In supplying a patient with an appliance of this kind we must secure correct impressions; then perfect articulating models; select teeth that are temperamentally suitable for the case in hand; when we must individualize and arrange the teeth; after we have moulded and carved the gum portion to restore the contour of the soft tissues we can reproduce all in permanent form, and we have the work ready for the practical test.

I have named six steps, no one of which can be said to be of less importance than the others. If we examine each step we will see that the mechanical and artistic are well blended: Impression, mechanical; articulations, mostly mechanical, a little of art in the rough; selection of teeth, purely art; arranging teeth, art and mechanics, half and half; carving gum, art; reproducing in permanent form, mechanical.

It is not our intention to discuss methods and materials in this paper, so we desire only to impress the importance of mechanically correct impressions.

The next important procedure is to secure correct articulating models. These are as essential to the artist in prosthesis as the clay model is to the sculptor. The method described by our text-books of a base plate with a rim of wax built to contour of teeth and gums is the only true method. The idea of the "mush-bite," so called, is preposterous.

The selecting of the teeth is truly an artistic operation, but I will admit, as it is usually done, is a most inartistic procedure. It is artistic because it is primarily a mental process assisted by the eye. It is mental, because we presume the operator analyzes the case and concludes what the general form, size and color of the teeth should be. This must be accomplished by a thorough knowledge of the temperaments. The eye must be trained in color, proportion and harmony. Harmony is the one attribute that marks the true artist; without it being either inherent or acquired it is impossible for a man to be a successful prosthetist.

It is absurd to talk about reproducing nature as the height of art in any prosthetic operation. Nature is so hedged about by environments that she is constantly producing monstrosities of greater or less degree. True art in prosthesis is producing what nature should have accomplished—that is, a harmonious whole. Otherwise there would be no need for the orthopedist in the dental profession devoting his entire energies to correcting congenital departures from the perfect harmony designed by the great Author of our being. Why should we insist that because our patient did not go to a successful orthopedist in his youth he shall continue the remainder of his days in as nearly the same abnormal condition as it is possible for art to serve him? We can modify and thereby harmonize all the associate parts and hence demonstrate we possess the true artistic sense. This is not a plea for undersize, white china, made in a mold, universal denture for all mankind; but rather that cultivated, refined dental art which we believe all thinking men must concede is the most difficult and should be the most exalted department of our profession.

I am glad to learn through our dental dealers that in many sections of our country the gum section teeth are largely out of use; that not more than one set is used to-day where five sets were used fifteen years ago. May this good work go on until their manufacture shall cease. Some worthy members of our profession condemn all teeth made in molds, and would require a dentist to carve the teeth. I do not believe in this ultraism; there are too few artists in the world to supply the demands of prosthesis, and very practical results can be obtained where we have a large stock of single or plain teeth from which to select. The same argument might be used in regard to the sculptor, that he should quarry his own marble. The dentist should select his stock teeth as the sculptor does his block of marble. The marble must contain the inherent qualities that will permit the sculptor to produce the beautiful and finished piece of art he desires. So the dentist must select the stock teeth that contain general form, size and color that will permit the dental artist to grind, shape, polish and perhaps stain to individualize and, so to speak, vitalize them.

Arranging the teeth—we have remarked that art and mechanics are equally essential in this operation. If we have the knowledge of art implied in selecting the teeth, we certainly ought to apply it in arranging the teeth. Then in the mechanical arranging of the teeth we must give attention to the laws of leverage or the denture will be a failure from the practical point of view.

Carving the gum portion, or restoring the contour of the soft tissues is of nearly as much importance as selecting and arranging the teeth. We have very little written and hear little said upon this subject, but it is the cause of many failures, considered æsthetically. To comprehend the great change produced by the loss of the teeth, and how to remedy the defect, we must know the changes in the hard tissues and the muscles of expression.

The alveolar process is designed by nature as the rigid support for the teeth, and when the teeth have been lost nature proceeds to remove by absorption that process, until in time there is hardly a vestige of it left. We can readily understand that any muscles attached to the changing hard tissues must be more or less affected, and that when this change consists of a wasting of the substance, or putting the muscle upon a tension, it is hardly in the province of the appliance, the office of which is to pad out, to rectify. We must conceive that there are limitations to our ability to restore, and we must use care not to exaggerate the defect. It is fortunate there are but few muscles attached to the bones that undergo resorption, but enough, however, to permanently alter the expression of the face. The larger portion of .the muscles of expression are under the control of the prosthetist.

Dr. Case informs us that the area of the face susceptible of change by the orthopedist is described by a line drawn from the lower border of the nasal bone, just below the malar process, turning downward about the anterior border of the ramus of the mandible, then continuing forward to the point of the chin. This he designates the changeable portion, and the rest of the face as the unchangeable or fixed portions. I desire to call attention to the fact that this includes the whole of the cartilaginous portion of the nose.

It is this changeable portion, and the whole of it, with which the prosthetist has to deal.

Resorption of the superior alveolar process causes the ridge to disappear as rapidly as the labial and buccal walls. The same principle holds true in regard to the inferior alveolar process.

There are eleven pairs and one single muscle that must be considered in the study of expression; several of these only need be mentioned, while others will require greater consideration.

The *orbicularis oris* is a constrictor muscle and has no bone attachments, so the only consideration we have to give it in contouring a denture is that it shall be held in an easy and graceful position, neither too tense nor relaxed.

Of the nasal group of muscles, the *compressor naris* arises in the upper part of the incisive fossa and is spread out upon the wing of the nose; its function is to dilate the nostrils. When the alveolar process recedes it carries the origin of the muscle farther away from its insertion and puts it upon a constant tension, hence constantly dilating the nose. This condition we can not overcome, but we should not add to the defect by making the plate so high and full at this point that it will increase the tension of this troublesome little muscle. The *depressor alæ nasi* arises in the incisive fossa lower down upon the alveolar ridge, divides it into two portions, ascending and descending; the ascending spreading out upon the septum and tip of the nose; hence the loss of the natural teeth causes a constant drawing downward of the tip of the nose. The descending portion unites with its mate of the opposite side, attaching to the under side of the *orbicularis*, forming the *frænum labia*. The descending portion is sometimes named *depressor labia sup*. This name describes its action, and reminds us of the unfortunate effect of the loss of the teeth, and explains why the plate must be notched at this position.

The next four muscles we can group together. *Levator labia . superioris Alæque nasi*, *levator labii superioris proprius*, *zygomaticus major* and *minor* arise respectively from the nasal process of the *sup. maxilla*, lower margin of the orbit, and the molar bane. They are all inserted near the angle of the mouth into the *orbicularis*. The first draws the corner of the mouth upward and forward, giving the expression of contempt. The second draws the corner of the mouth upward, and the third and fourth upward and backward, used in laughing. These are important muscles of expression, but are not difficult to control, because their origins are relatively a long distance from their insertions.

The *levator anguli oris* is a more important muscle arising from the cuspid fossa and inserted in the *orbicularis* at the angle of the mouth. A portion of this muscle forms the buccal frænum. The *buccinator*, or bugle blower's muscle, arising from the alveolar process over the molars of the superior maxilla and is inserted in the corresponding portion of the mandible. This muscle is most decidedly affected by the extraction of the teeth and it is impossible to perfectly restore its contour, and sometimes an attempt in this direction will result in dislodging the denture.

The *depressor anguli oris* arises from the oblique line of the lower jaw and is inserted into the *orbicularis oris* at the angle of the mouth.

The *depressor labii inferioris*, or *quadratus menti*, arises inside of the preceding muscle and extends toward the symphisis and is inserted into the *orbicularis oris* throughout the lower lip. Often a portion of this muscle arises higher upon the alveolar process in the location of the cuspid fossa, producing an inferior buccal frænum. Because of the excessive development and high attachment upon the alveolar process of this muscle it is often very troublesome to so contour the lower denture as to produce a pleasing expression.

The *levator labii inferioris* arises from the inferior incisive fossa and unites with its fellow of the opposite side, forming the frænum of the upper lip, which must always be provided for in contouring the plate.

The *platysma myoides* and other muscles are also considered muscles of expression, but are of no consequence to the prosthetist.

A study of these twelve muscles named will aid in giving us an intelligent idea of the required contour of the gum portion of an artificial denture. It is also necessary that we study in connection with the muscles the surface markings of the alveolar process while the teeth are in situ.

The most prominent marking is the cuspid eminence immediately over the cuspid tooth, and directly under the *levator superioris proprius*, and is necessary for the contour of this muscle. Immediately back of the cuspid eminence is the cuspid fossa, occupied by the *levator anguli oris* muscle. In restoring the cuspid eminence it must be carried as high as possible, then dropped abruptly to accommodate the angle muscle and represent the fossa.

In front of the cuspid eminence is the incisive fossa, most marked over the lateral incisor. If we do not represent this fossa we exaggerate the effect of the muscle of contempt, dilate the nostrils, depress the tip of the nose, and give a bulging curve to the lip in the median line.

It has sometimes been said that all there is in giving contour and expression to an artificial denture is to produce the cuspid eminences. True, this is a very important and universal feature, and any soul, no matter how devoid of artistic sense, can see this necessity; but if we desire to be classed as artists indeed, we must continue our studies and untiring efforts until we can work with the great Creator in producing the "human face divine."

DISCUSSION.

Dr. G. Molyneaux: The paper presented by Dr. Wilson is certainly one worthy of a most careful and serious consideration. If the prosthetic department of dentistry is ever credited with the noble position to which it is entitled, it will only come through a rational consideration of the subject. A careful study of the underlying principles of prosthetic dentistry, their promulgation, and subsequent exemplification by such appliances as shall best meet the desired end, is the only hope. We too often expect our appliance to counteract our ignorance of the subject. If we err in judgment through ignorance, no apparatus will make our work successful. The abuse of this department comes largely through ignorance of its principles and a devotion to mechanics or the jewelry work of dentistry.

The essayist has listed prosthetic dentistry as a department of stomatology, which I do not like. He has objected to prosthodontia, and I agree with him in his reasons for not accepting a term that carries with it no special significance. Stomatology has no place in dentistry at present. It is specialized by the medical profession, and refers by various constructions to the word *stoma*, meaning an opening, to pathological conditions of the pores or cells of the mucous membrane lining the mouth and pharynx, and is not used in any other sense except by a few dentists. Prosthetic dentistry is a department of the practice of dentistry, and the essayist refers to it as such, except in his prefatory statement. Instead of dividing the practice of dentistry into departments with obscure nomenclature, let us classify the division under consideration itself. The essayist differentiates operative dentistry as chair work and prosthetic dentistry as bench work ; so long as this distinction exists so long will prosthetic dentistry occupy the very narrow sphere suggested by the essayist. Dental prosthesis is one thing, the exemplification of dental prosthesis through dental mechanics is another. Dental prosthesis and dental mechanics are divisions that must sooner or later come into general use.

A good impression, the essayist says, is the foundation of the denture ; I say the same. But how many of us take what we think a good impression, because it resists the effort of removal, and think the problem is solved. Unless an impression represents the tissues covered in their natural position of repose, every inequality of the ridge, or palate, every duplication of mucous membrane defining the limitation of base of denture without trimming, it is not a good impression no matter how forcibly it resists the effort of removal. It is impossible for me to attempt a full discussion of the matter of impressions of the mouth, articulation of teeth, angles of force, etc., at this time, but I do hope to present some thought on these lines very soon. Art in dentistry, so far as cosmetic uses require, is not essential. It is, however, expected to be coupled with other good qualities of the denture. The essayist says true art in prosthesis is producing what nature should have accomplished, but this is apt to be misleading unless qualified. There are times when dealing with young patients that we resort to mechanism for cosmetic purposes, when orthopedic dentistry is out of the question. But when we are dealing with people past middle life, who are associated with certain unusual characteristics, such as protruded lower or upper maxilla, and these patients have lost their teeth, requiring artificial substitutes, we should not wholly eradicate this characteristic expression. A restoration of the natural expression is art, but to make a patient look like some one else is not art. We are not all capable of expressing art from a cosmetic standpoint in artificial dentures. Environment, clientele and diverse other matters prevent this true expression. We are not, however, excused from ignoring the laws of hygiene in making a denture.

The essayist refers to a wasting disease of the alveolar ridges. Have we not solved the cause of this trouble? Is it not possible that under certain conditions natural teeth are retained too long? Is it not possible that many times a poorly constructed denture is the cause of resorption of the ridges by functional interference? Who is to solve these questions of so much importance, the bench workman or the intelligent dentist? The prosthetic department of dentistry can not at this time be divorced from the guiding integrity of the observing dentist at the chair. If we turn our patients over to the laboratory man to have impression taken and work done, we can not expect very much for the denture. The intelligent dentist must guide the mechanic and start him, and must see that everything is carried out correctly, must judge as to the best means of protecting mouth, preserving ridges, if possible, for the time being, as the wasting of the process is an important question. This work, it seems to me, is just as truly ennobling as any other part of dentistry.

DR. H. M. KIRK: It was said several years ago that in the study of dentistry the mechanical side was the basis. This is true, I think. Mechanical skill and mechanical ingenuity are largely a mental development; no matter how much dexterity you have in the fingers or how much manipulative skill, unless they are associated with good judgment and proper knowledge ot what is best for each case, they are useless.

So I think the Doctor was correct when he said that the same mental and manipulative faculties were used in both mechanical and operative dentistry.

I will agree with the essayist that prosthetic dentistry is a largely abused and neglected department of dentistry, and there is no reason why this should be. It is just as lawful, just as honorable and just as noble as operative, and it is also just as remunerative, and there there is no reason why it should be considered less in degree than operative. Of course, prosthetic dentistry is laboratory work, but it is easier than operative, can be done at odd times and while sitting down; but yet many dentists say they do not like the laboratory work, while at the same time they are more than glad to go into the laboratory and work on crowns and bridges, and they are highly elated to have all this work they can get, although it entails working in the laboratory.

I think the true reason why prosthetic dentistry is neglected and abused is because the dentist is unsuccessful in the building up of dentures and in their proper adaptation to the mouth. They lack this mental development. There are certain principles of mechanical laws which are involved in the setting up and arrangement of teeth, in the articulation, in the trimming of the plates, and whether or not there should be a relief chamber, etc., all very important and overlooked by a large number of dentists. For these reasons they are unsuccessful, and therefore get no enjoyment out of the work.

The essayist speaks of gum teeth—of their not being so much in use as some years ago. We all know the advantages and disadvantages of gum teeth. Occasionally they are very useful, but they have been very greatly displaced by the plain teeth. The reason, I think, is because of their poor shape and difficulty of putting them up in a natural looking denture. Dr. Haskell said: "The reason teeth are not of better shape is because the profession do not demand it. If they wanted better forms they would be manufactured for them." So it seems the dentists are to blame. The bicuspids and the molars are the greatest offenders; they are not broad enough on the grinding surface, and it is impossible to Bonwill the teeth. It is wonderful what we can do in restoring the expression and intelligence of the faces of our patients, but as long as dentists will continue to make plates and set up teeth without any attributes of the natural teeth prosthetic dentistry will hold the place it now has. It is true that the majority of people will flock to these sign-emblazoned offices and to these modern dental monstrosities, but we must educate them to the importance and value of good work in prosthetic dentistry.

Dr. Ruggles: There is one subject which comes up in connection with gum and plain teeth. I would like to ask which is the better to use in setting up plates. In the plain teeth we can by grinding them in different ways set them up to look irregular and more natural, while with gum teeth we are compelled to grind them in only the one general way. Now, which is the greater disadvantage: to have the rubber showing and the teeth set up irregularly to look more natural, or have the teeth set up perfectly regular with the porcelain gum?

Dr. Kirk: In reference to Dr. Ruggles' statement, there are some cases where we are compelled by the nature of the case, the shape of the mouth, shortness of upper lip and length of gum, to use gum teeth. If this is not necessary, the use of plain teeth is advised. So I should say, use gum teeth only in those cases where we can not use plain.

Dr. Wilson; I think if we all understood each other perfectly there would be very little difference in the understanding of prosthetic dentistry between the discussors and myself. It is always the object of the first man who speaks to create a discussion, and I think that he made a few stronger points in some respects than he really believes. One point, he said that we should supply what nature should do, restore the natural expression. He immediately followed that statement by the very expression I used in the paper, "we are not to follow nature, but make them look like their natural self." Nature changes from year to year, so a patient becomes an unsightly creature after a while—shall we keep them in that same condition? There is no thought of art in that at all. I make no plea of having an universal set for each person. And an ideal form is not what I mean, but to harmonize. If the teeth are all out of alignment, due to extractions, pyorrhea, etc., the work of bad operating, probably, should we keep them in that same condition? (That is, restoring their natural expression?)

Not at all, nor should we have a set of teeth representing patients sixteen years of age when they are sixty. The thought of harmony should pervade the whole subject of prosthetic dentistry, otherwise there is no art in it.

In reference to the term "prosthodontia," I don't like riveting our minds completely upon the teeth. When we, as dentists, can see beyond the teeth, then we can call ourselves professional men; but when we have that one narrow thought of teeth, we are simply mechanics. The whole facial expression, etc., is to be considered; that is the reason I don't like "prosthodontia." "Prosthetic dentistry" is also just as bad. *Stoma* is used to mean the mouth; the first thought is probably of the stomach, and when we learn it means the mouth it is not pleasing at all, but I think it is better than any word we have at the present time. We call it "stomatology." It covers the whole thought of the mouth and its surroundings, gums, palate, tongue, nose, etc. I think the word is much better than the terms above mentioned, and must use it until some word which is more appropriate is found. I grant that it is not euphonious at all.

---

DISINFECTION OF THE HANDS FOR SURGICAL OPERATIONS.—Wash the hands carefully with soap and warm soft water, clean and pare the nails, wash again in new water, and then in a nine percent solution of essence of cinnamon or a twelve percent solution of essence of thyme.—*Eugenio Calvello.*

